# Synthesis and antioxidant activities of new nickel(II) complexes derived from 4-benzyloxysalicylidene-S-methyl/propyl thiosemicarbazones

**DOI:** 10.3906/kim-2101-12

**Published:** 2021-06-30

**Authors:** Songül EĞLENCE-BAKIR

**Affiliations:** 1 Department of Chemistry, Faculty of Science, İstanbul University, İstanbul Turkey

**Keywords:** Thiosemicarbazone, nickel(II) complex, structural analysis, antioxidant capacity, radical scavenging activity

## Abstract

Six nickel(II) complexes of the N_2_O_2_ chelating thiosemicarbazones were synthesized using N^1^-4-benzyloxysalicylidene-S-methyl/propyl thiosemicarbazone and methoxy-substitute-salicylaldehydes in the presence of Ni(II) ion by template reaction. The structures of thiosemicarbazones and nickel(II) complexes were characterized by elemental analysis, UV-Vis, IR, and ^1^H-NMR spectroscopies. The structure of the N^1^-4-benzyloxysalicylidene-S-propyl thiosemicarbazone ( **2** ) was determined by X-ray single-crystal diffraction method. The total antioxidant capacities of synthesized compounds were evaluated by using cupric reducing antioxidant capacity (CUPRAC) method. The thiosemicarbazones exhibited more potent antioxidant capacity than Ni(II) complexes. Trolox equivalent antioxidant capacity (TEAC) of **1c** was found highest in tested nickel(II) complexes. In addition, antioxidant activities of tested compounds were evaluated by using the hydroxyl radical, DPPH radical, and ABTS radical scavenging abilities of these compounds.

## 1. Introduction

Thiosemicarbazide, N^1^H_2_–NH–C(=S)–N^4^H_2_, is an important precursor used in the synthesis of many polydentate ligands [1–3]. The reactivity of its two terminal amino groups differ from each other [4]. The amino functional group of the molecule (N^1^H_2_-) is more reactive and easily condenses with the 2-hydroxy/amino substitute carbonyl compounds to form schiff bases [5–7]; whereas, the thioamidic group (-N^4^H_2_) has a very weak potential to react with carbonyl compounds. However, by performing a double-condensation reaction in the presence of transition metal ions having template effect and a second carbonyl molecule, thiosemicarbazone-metal complexes with tetradentate coordination can be obtained [8–10]. These compounds were first synthesized and characterized in 1971 [11]. 

**Figure 1:**
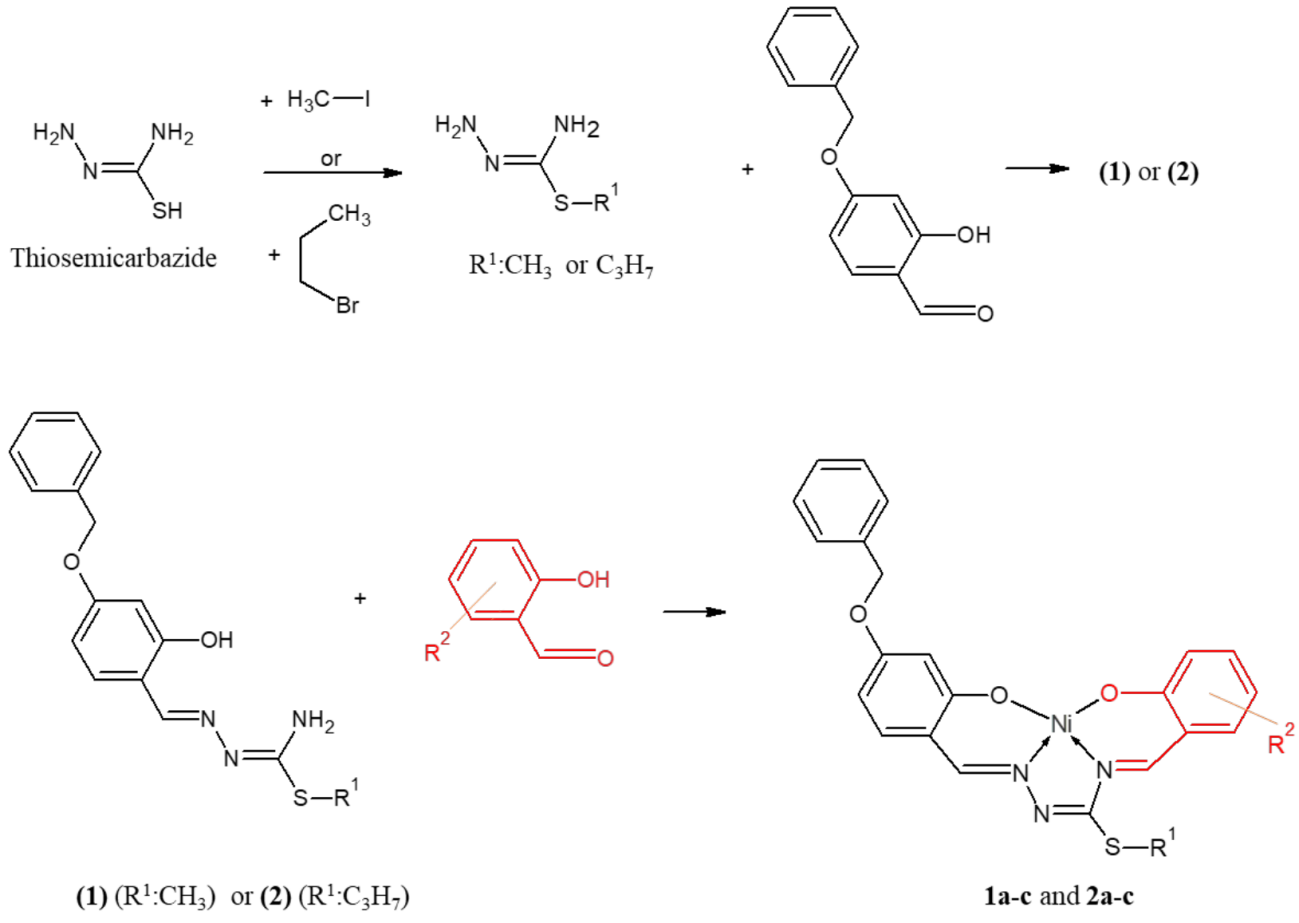
Synthesis scheme for the nickel(II) complexes. 1a : (R1:CH3; R2: 3-OCH3); 1b: (R1:CH3; R2: 4-OCH3); 1c: (R1:CH3; R2: 5-OCH3) 2a: (R1:C3H7; R2: 3-OCH3); 2b: (R1:C3H7; R2: 4-OCH3); 2c: (R1:C3H7; R2: 5-OCH3)

The biological activities of thiosemicarbazones and their metal complexes have been known for a long time [12–15]. In addition, there is a significant amount of published studies describing the role of their uses as sensors [16,17], optics [18], and catalysts [19]. Many studies have revealed that the activities depend on the type of metal and the substituted groups attached to hydrazine, amide and sulfur atoms [20,21]. Therefore, it becomes important to systematically synthesize and examine compounds with different groups on both the aromatic ring and the amide and sulfur atoms. When the biological activity studies of thiosemicarbazone and metal complexes are examined, studies containing antioxidant activity are remarkable. In studies examining the antioxidant activities of thiosemicarbazones and nickel (II) complexes containing –OH groups in the aromatic rings, 3/4–hydroxy salicylaldehyde or 2,2′-dihydroxybenzophenone, it was observed that the activity was high due to the presence of these groups [22–24]. In order to examine how methoxy and benzyloxy groups affect the antioxidant activity; we synthesized six new nickel(II) complexes (Figure 1) using N^1^-4-benzyloxysalicylidene-S-methyl/proyl-thiosemicarbazones and 3-, 4-, and 5-methoxy salicylaldehydes. The structure of the N^1^-4-benzyloxysalicylidene-S-propyl thiosemicarbazone ( **2** ) has been determined by X-ray diffraction method. Besides, we examined the antioxidant capacities and radical scavenging activities of the free thiosemicarbazones and nickel(II) complexes in vitro assays for summarize their biochemical properties. 

## 2. Materials and methods

### 2.1. Materials and physical measurements

The chemicals used were of reagent grade and used without purification. Analytical data were determined on a Thermo-Finnigan Flash EA 1112 Series Elementary Analyzer. Electronic spectra of the compounds were recorded at 5×10^–5^ M concentration in CHCL_3 _solution with a Shimadzu 2600 UV-Vis Spectrometer. Infrared spectra were obtained on an Agilent Carry 630 FTIR-ATR spectrophotometer in range of 4000 to 400 cm^−1 ^at room temperature. ^1^H-NMR spectra were performed on a Bruker Avance-500 model spectrometer relative to SiMe_4_. The X-ray data were recorded on a Bruker APEX II QUAZAR three-circle diffractometer.

### 2.2. Synthesis of thiosemicarbazones

The thiosemicarbazones were obtained according to literature methods with small modifications [25], and synthesis procedure was described as follows by the N^1^-4-benzyloxysalicylidene-S-methylthiosemicarbazone ( **1** ) as representative example. 0.5 g (5 mmol) thiosemicarbazide and 0.33 mL (5 mmol) methyl iodide were refluxed in 10 mL ethanol for 2 h. Three milliliters of ethanolic solution of 4-benzyloxysalicylaldehyde (1.25 g, 5 mmol) was added. The mixture was refluxed two more hours and allowed to stand at room temperature overnight. The separated yellow precipitate was filtered, washed several times with cold ethanol and dried under vacuum. The N^1^-4-benzyloxysalicylidene-S-propyl thiosemicarbazone ( **2** ) was obtained by using 1-bromopropane (5 mmol, 0.45 mL) with the same procedure. Hydrohalide forms of the thiosemicarbazones were used in the synthesis of complexes. The colors, m.p. (°C), yields (%), elemental analyzes, UV-Vis, IR and ^1^H-NMR data of the thiosemicarbazones are given as follows:

**N** **^1^** **-4-benzyloxysalicylidene-S-methylthiosemicarbazone (1):** Light yellow, 134°C, 63%, Anal. Found (Calc.) for C_16_H_18_IN_3_O_2_S (443.30 g/mol) C, 43.21 (43.35); H, 4.19 (4.09); N, 9.33 (9.48); S, 7.65 (7.53)%. UV-Vis (nm, log ε in parenthesis): 240 (2480); 294 (2300); 305 (2840). IR (ATR, cm^-1^): *υ* (OH) 3457; *υ*_s_(NH_2_) 3281; ʋ(CH) *_aliph._* 2786, 2646; *υ* (C=N^1^) 1602. ^1^H NMR (CDCl_3_): 11.53, 11.31 (s, 1H, i:2/1, *OH* ); 8.33, 8.20 (s, 1H, i:2/1 *CH=N* *^1^* ); 7.39-7.07 (m, 8H, *aromatics* ); 6.51 (m, 2H, *NH* *_2_* ); 5.03 (s, 2H, O *-CH* *_2_* ); 2.48 (s, 3H, S-C *H* *_3_* ).

**N** **^1^** **-4-benzyloxysalicylidene-S-propylthiosemicarbazone (2):** Light yellow, 115°C, 66%, Anal. Found (Calc.) for C_18_H_22_BrN_3_O_2_S (424.35 g/mol) C, 50.81 (50.95); H, 5.19 (5.23); N, 9.73 (9.90); S, 7.65 (7.56)%. UV-Vis (nm, log ε in parenthesis): 240 (6900); 294 (6440); 305 (7800). IR (ATR, cm^-1^): *υ* (OH) 3365; *υ*_s_(NH_2_) 3332; ʋ(CH) *_aliph._* 2879, 2791; *υ* (C=N^1^) 1539. ^1^H NMR (CDCl_3_): 11.76, 11.56 (s, 1H, i:1/2, *OH* ); 8.31, 8.18 (s, 1H, i:2/1 *CH=N* *^1^* ); 7.39-7.06 (m, 8H, *aromatics* ); 6.50 (m, 2H, *NH* *_2_* ); 5.01 (s, 2H, O *-CH* *_2_* ); 3.00, 2.98 (t, 2H, i:3/1, J:7.32 *S-CH* *_2_* *-* ); 1.68 (m, 2H, *-CH* *_2_* *-* ); 1.02, 0.81 (t, 3H, J:7.32, i:2/1, - *CH* *_3_* ). 

### 2.3. Synthesis of the complexes

The nickel(II) complexes were obtained according to literature methods with small modifications [6], and synthesis procedure was described as follows by the N^1^-benzyloxysalicylidene-N^4^-3-methoxysalicylidene-S-methylthiosemicarbazidato nickel(II) ( **1a** ) as representative example. N^1^-4-benzyloxysalicylidene-S-methylthiosemicarbazone (0.44 g, 1 mmol) and 3-methoxysalicylaldehyde (0.15 g, 1 mmol) were dissolved in 25 ml warm ethanol and added to the ethanolic solution of nickel(II) chloride hexahydrate (0.23 g, 1 mmol) by stirring. The mixture was stirred at 40 °C for two hours and than allowed to stand at room temperature for a few days. The red product was collected by filtration and washed with cold ethanol. The crystalline powders were dried over P_2_O_5_ under vacuum. The other nickel complexes were synthesized with the same procedure described above using N^1^-4-benzyloxysalicylidene-S-propylthiosemicarbazone and 4-/5-methoxy substitue salicylaldehydes. The colors, m.p. (°C), yields (%), elemental analyzes, UV-Vis, IR and ^1^H-NMR data of the nickel(II) complexes are given as follows:

**N** **^1^** **-benzyloxysalicylidene-N** **^4^** **-3-methoxysalicylidene-S-methylthiosemicarbazidato nickel(II) (1a):** Red, 270 °C, 72%, Anal. Found (Calc.) for C_24_H_21_N_3_NiO_4_S (506.20 g/mol) C, 56.74 (56.95); H, 4.31 (4.18); N, 8.19 (8.30); S, 6.38 (6.33)%. UV-Vis (nm, log ε in parenthesis): 240 (10860); 294 (10520); 305 (12960); 505 (6040). IR (ATR, cm^-1^): ʋ(CH) *_aliph._* 2935, 2916; *υ* (C=N^1^) 1599; *υ* (C=N^2^) 1530.^1^H NMR (DMSO-d_6_): 8.39, (s, 1H, *CH=N* *^1^* ); 8.20, (s, 1H, *CH=N* *^4^* ); 7.48-6.40 (m, 11H, *aromatics* ); 5.15 (s, 2H, O *-CH* *_2_* ); 3.84 (s, 3H, O *-CH* *_3_* ); 2.70 (s, 3H, S- *CH* *_3_* ). 

**N** **^1^** **-benzyloxysalicylidene-N** **^4^** **-4-methoxysalicylidene-S-methylthiosemicarbazidato nickel(II) (1b):** Red, 284°C, 70%, Anal. Found (Calc.) for C_24_H_21_N_3_NiO_4_S (506.20 g/mol) C, 56.81 (56.95); H, 4.27 (4.18); N, 8.22 (8.30); S, 6.26 (6.33)%. UV-Vis (nm, log ε in parenthesis): 242 (7700); 295 (7920); 305 (9720); 499 (5880). IR (ATR, cm^-1^): ʋ(CH) *_aliph._* 2901, 2866; *υ* (C=N^1^) 1539; *υ* (C=N^2^) 1500.^1^H NMR (DMSO-d_6_): 8.34, (s, 1H, *CH=N* *^1^* ); 8.06, (s, 1H, *CH=N* *^4^* ); 7.65-6.39 (m, 11H, *aromatics* ); 5.14 (s, 2H, O *-CH* *_2_* ); 3.81 (s, 3H, O *-CH* *_3_* ); 2.68 (s, 3H, S- *CH* *_3_* ). 

**N** **^1^** **-benzyloxysalicylidene-N** **^4^** **-5-methoxysalicylidene-S-methylthiosemicarbazidato nickel(II) (1c):** Red, 269°C, 69%, Anal. Found (Calc.) for C_24_H_21_N_3_NiO_4_S (506.20 g/mol) C, 57.01 (56.95); H, 4.31 (4.18); N, 8.44 (8.30); S, 6.49 (6.33)%. UV-Vis (nm, log ε in parenthesis): 242 (12260); 294 (12540); 305 (15460); 510 (6800). IR (ATR, cm^-1^): ʋ(CH) *_aliph._* 2891, 2872; *υ* (C=N^1^) 1599; *υ* (C=N^2^) 1517.^1^H NMR (DMSO-d_6_): 8.33, (s, 1H, *CH=N* *^1^* ); 8.07, (s, 1H, *CH=N* *^4^* ); 7.44-6.27 (m, 11H, *aromatics* ); 5.10 (s, 2H, O *-CH* *_2_* ); 3.72 (s, 3H, O *-CH* *_3_* ); 2.65 (s, 3H, S- *CH* *_3_* ). 

**N** **^1^** **-benzyloxysalicylidene-N** **^4^** **-3-methoxysalicylidene-S-propylthiosemicarbazidato nickel(II) (2a):** Red, 197°C, 66%, Anal. Found (Calc.) for C_26_H_25_N_3_NiO_4_S (534.25 g/mol) C, 58.31 (58.45); H, 4.63 (4.72); N, 8.01 (7.87); S, 6.19 (6.00)%. UV-Vis (nm, log ε in parenthesis): 244 (7640); 294 (7880); 305 (9780); 522 (5160). IR (ATR, cm^-1^): ʋ(CH) *_aliph._* 2927, 2861; *υ* (C=N^1^) 1536; *υ* (C=N^2^) 1498.^1^H NMR (DMSO-d_6_): 8.39, (s, 1H, *CH=N* *^1^* ); 8.28, (s, 1H, *CH=N* *^4^* ); 7.49-6.41 (m, 11H, *aromatics* ); 5.15 (s, 2H, O *-CH* *_2_* ); 3.75 (s, 3H, O *-CH* *_3_* ); 3.28, (t, 2H, J:0.015, *S-CH* *_2_* *-* ); 1.77 (m, 2H, *-CH* *_2_* *-* ); 1.01 (t, J:0.014 3H, - *CH* *_3_* ). 

**N** **^1^** **-benzyloxysalicylidene-N** **^4^** **-4-methoxysalicylidene-S-propylthiosemicarbazidato nickel(II) (2b):** Red, 186°C, 53%, Anal. Found (Calc.) for C_26_H_25_N_3_NiO_4_S (534.25 g/mol) C, 58.51 (58.45); H, 4.59 (4.72); N, 7.81 (7.87); S, 5.86 (6.00)%. UV-Vis (nm, log ε in parenthesis): 244 (9860); 294 (9878); 306 (12460); 509 (7860). IR (ATR, cm^-1^): ʋ(CH) *_aliph._* 2926, 2866; *υ* (C=N^1^) 1597; *υ* (C=N^2^) 1590.^1^H NMR (DMSO-d_6_): 8.31, (s, 1H, *CH=N* *^1^* ); 8.08, (s, 1H, *CH=N* *^4^* ); 7.63-6.37 (m, 11H, *aromatics* ); 5.13 (s, 2H, O *-CH* *_2_* ); 3.81 (s, 3H, O *-CH* *_3_* ); 3.24, (t, 2H,J:0.015, *S-CH* *_2_* *-* ); 1.72 (m, 2H, *-CH* *_2_* *-* ); 1.00 (t, 3H, J:0.017, - *CH* *_3_* ). 

**N** **^1^** **-benzyloxysalicylidene-N** **^4^** **-5-methoxysalicylidene-S-propylthiosemicarbazidato nickel(II) (2c):** Red, 205°C, 51%, Anal. Found (Calc.) for C_26_H_25_N_3_NiO_4_S (534.25 g/mol) C, 58.61 (58.45); H, 4.79 (4.72); N, 7.91 (7.87); S, 6.11 (6.00)%. UV-Vis (nm, log ε in parenthesis): 244 (9860); 294 (9870); 306 (12460); 511 (7860). IR (ATR, cm^-1^): ʋ(CH) *_aliph._* 2928, 2866; *υ* (C=N^1^) 1541; *υ* (C=N^2^) 1490.^1^H NMR (DMSO-d_6_): 8.37, (s, 1H, *CH=N* *^1^* ); 8.26, (s, 1H, *CH=N* *^4^* ); 7.48-6.39 (m, 11H, *aromatics* ); 5.13 (s, 2H, O *-CH* *_2_* ); 3.71 (s, 3H, O *-CH* *_3_* ); 3.28, (t, 2H, J:0.015, *S-CH* *_2_* *-* ); 1.74 (m, 2H, *-CH* *_2_* *-* ); 1.01 (t, 3H, J:0.014, - *CH* *_3_* ). 

### 2.4. Antioxidant property analysis

Different spectrophotometric assays were performed to evaluate the antioxidant properties of N^1^-4-benzyloxysalicylidene-S-methyl/propyl-thiosemicarbazones and nickel(II) complexes. CUPRAC (cupric reducing antioxidant capacity) method [26, 27] was used for total antioxidant capacity determination. The CUPRAC method is based on the reduction of a Cu (II) -Nc complex to a yellow-orange colored Cu (I) -Nc solution in the presence of antioxidants and a measurement of absorbance at 450 nm. Results were reported as trolox equivalent antioxidant capacity (TEAC). The free radical scavenging activities of synthesized compounds were determined by the DPPH method [28]. The DPPH activities were evaluated with respect to inhibition percentage of DPPH radical at 517 nm after reaction with a given samples. ABTS (2,2’- azinobis-(3-ethylbenzothiazoline-6-sulfonic acid))/persulfate method [29] was used to measure the antioxidant activity of synthesized compounds. This method is based on the inhibition of absorbance of the chromogenic radical cation of ABTS (ABTS **^.^** ^+^) by antioxidants. Antioxidant activity was determined as a percentage inhibition of ABTS **^.^** ^+^ with respect to the decrease in the absorbance of the ABTS **^.^** ^+^ within a certain period of time in the presence of antioxidants. Hydroxyl radicals in the aqueous medium were produced through the Fenton system, and the hydroxyl radical scavenging (HRS) activities of the samples were determined using the modified CUPRAC method [30]. The initial concentration of each sample in the ABTS **^.^** ^+^ scavenging, DPPH scavenging and HRS activity determinations were used to be 10^-3 ^M. In these tests, 0.5 mL were taken from the samples for all three methods. All antioxidant tests were carried out in triplicate.

### 2.5. X-ray crystallography

**Figure 2:**
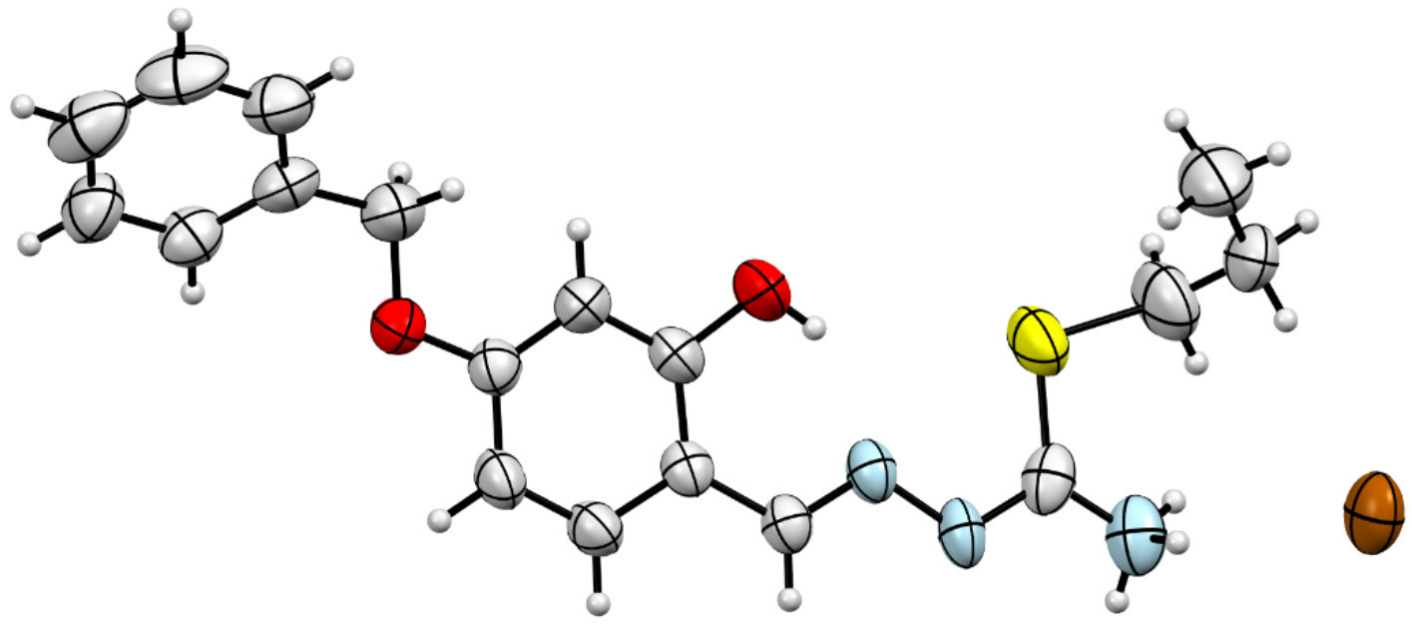
ORTEP diagram of compound 2 showing the labelling scheme of all atoms with 50% probability level.

**Figure 3:**
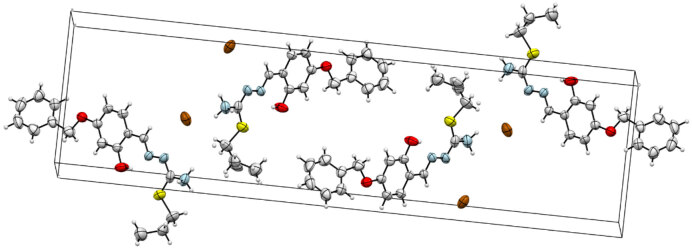
A partial packing diagram of the compound 2 in unit cell.

**Figure 4:**
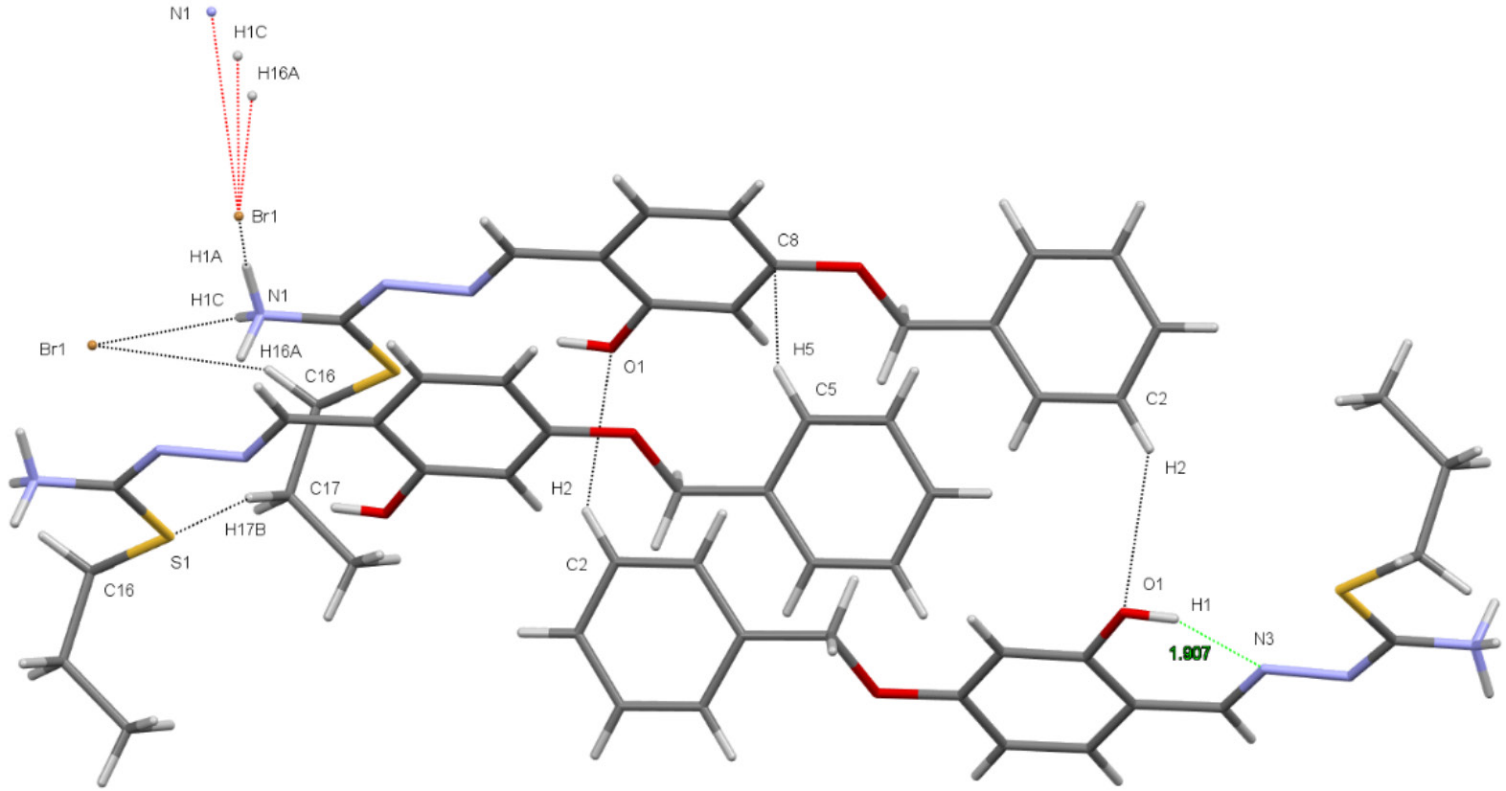
Hydrogen-bond network.

The crystals of **2** suitable for X-ray diffraction analysis were obtained by slow evaporation of an ethanol solution at room temperature. A light yellow crystal of 2, C_18_H_22_BrN_3_O_2_S, having approximate dimensions of 0.32 × 0.06 ×0.03 mm, was mounted on a glass fiber. All measurements were made on a Bruker APEX-II CCD imaging plate area detector with graphite monochromatic Mo-K αradiation (λ= 0.71073Å). Experimental conditions were summarized in Table 1. The crystal structure was solved and refined with SHELXTL [31]. The non-hydrogen atoms were refined anisotropically. H atoms were located in geometrically idealized positions C-H = 0.95(6) Å and treated as riding and *U* iso (H) = 1.2 *U* eq (C). The intra-intermolecular interactions were given in Table 2. Drawing was performed with the program ORTEP-III [32] with 50% probability displacement ellipsoid. ORTEP and unit cell packing diagram for 2 were given in Figures 2 and 3. The hydrogen-bond network was shown in Figure 4. Crystallographic data (excluding structure factors) for the structure in this paper has been deposited in the Cambridge Crystallographic Data Centre as supplementary publication number CCDC-2034512 for 2. Copies of the data can be obtained, free of charge, via www.ccdc.cam.ac.uk/conts/retrieving.html or from the Cambridge Crystallographic Data Centre, CCDC, 12 Union Road, Cambridge CB2 1EZ, UK; fax: + 44 1223 336033. E-mail: deposit@ccdc.cam.ac.uk.

**Table 1:** Crystal data and structure refinement for compound 2.

Parameters	
CCDC deposition no.	2034512
Chemical formula	C18H22BrN3O2S
Formula weight (g.mol-1)	424.35
Temperature (K)	296.15
Wavelength (Å)	0.71073
Crystal system	monoclinic
Space group	P21/c
Unit cell parameters	
a, b, c (Å)	4.9253(12), 38.615(10), 10.353(3)
b (°)	100.907(18)
Volume (Å3)	1933.4(8)
Z	4
Dcalc (g.cm-3)	1.458
Absorption correction	multi-scan
Tmin, Tmax	0.850, 0.937
F(000)	872.0
Crystal size (mm3)	0.327 x 0.060 x 0.029
Diffractometer/measurement method	
Index ranges h,k,l	-5<=h<=5, -42<=k<=45, -12<=l<=12
2θ Range for data collection (°)	6.330° < 2θ < 50.048°
Reflections collected	22042
Independent reflections	3416 [Rint = 0.1081, Rsigma = 0.0955]
Refinement method	Full-matrix least-squares on F2
Data/restraints/parameters	3416 / 21 / 239
Goodness-of-fit on F2	1.014
Final R indexes [I>=2σ (I)]	R1 = 0.0556, wR2 = 0.1236
R indices (all data)	R1 = 0.1474, wR2 = 0.1592

**Table 2:** Intra-intermolecular interactions (Å, °).

D—H····A	D—H	H····A	D····A	D—H····A
C2-H2····O1	0.930	2.710	3.47(1)	139.9
C5-H5····C8	0.931	2.849	3.42(8)	120.6
C17-H17B····S1	0.970	2.628	3.57(1)	164.9
C16-H16A····Br1	0.971	2.912	3.79(7)	151.8
N1-H1C····Br1	0.890	2.634	3.33(5)	135.4
N1-H1A····Br1	0.890	2.673	3.35(5)	133.3
*O1-H1····N3	0.821	1.907	2.63(6)	147.0

## 3. Results

### 3.1. Some physical properties of the compounds

The colour of S-alkylthiosemicarbazones, obtained as crystalline powders, are light yellow. They are soluble in common organic solvents such as alcohols, chloroform, dichloromethane, dimethyl sulfoxide etc. The red colored nickel(II) complexes are stable in the air. For thiosemicarbazones and nickel(II) complexes, the methyl substitute derivatives have higher melting point than those with propyl substitution. 

### 3.2. Spectral analysis

In the UV-Vis spectra of thiosemicarbazones the band observed at 240 nm is attributed to π→π* transition belonging to substitute benzene group. n→π* transitions associated with the imine group of thiosemicarbazone moiety are recorded at 294 and 305 nm [33, 34]. These bands were also detected at 240–244 nm and 294–306 nm in the spectra of the complexes. The metal→ligand charge transfer transitions were seen at 499–522 nm as weak absorption bands [22].

In the infrared spectra of thiosemicarbazones, the *υ* (OH) and *υ* (NH) bands were recorded at 3457, 3365 cm^–1^ and 3281, 3332 cm^–1^ for 1, 2 respectively. These bands were not present in the spectra of nickel(II) complexes because of the coordination of the phenolic oxygen and amidic nitrogen. The strong *υ* (C=N^1^) bands of compounds were observed at 1536–1602 cm^–1 ^range [35]. In the spectra of complexes, new azomethine bands, *υ* (C=N^4^), were monitored at 1490–1590 cm^–1 ^range due to the condensation of thioamide nitrogen and second aldehyde [36]. In addition, the aliphatic *υ* (CH) bands were observed at 2646–2935 cm^–1^ range [37].

When ^1^H-NMR spectra of thiosemicarbazones are examined, the proton of the phenolic hydroxyl group appeared as singlet at 11.53,11.31 ppm (for 1) and 11.76, 11.56 ppm (for 2) in different isomer ratios. The absence of these peaks in their complex spectra supports that the coordination is through phenolic oxygen [38]. In addition, the isomerism of the CH=N^1^ group, which was recorded as singlet in the range of 8.33–8.18 ppm in the spectra of thiosemicarbazones, was not observed in the spectra of the complexes due to hindered rotation around C=N^1^ and C=N^4^ double bond on complexation [39]. The peaks of the new CH=N^4^ group formed as a result of the template reaction were observed as singlets in the spectra of nickel(II) complexes in the range of 8.28–8.06 ppm [40]. Also, recording the peaks of methoxy substituted aldehyde at expected values supports its contribution in complex formation.

### 3.3. Crystallography

The single crystal of N^1^-4-benzyloxysalicylidene-S-propylthiosemicarbazone ( **2** ) were grown by slow evaporation of the ethanolic solution. The thiosemicarbazone crystallize in the monoclinic P21/c space group. The ortep diagram of the compound with displacement ellipsoids drawn at 50% probability level is given in Figure 2. Crystal data and structure refinement details are given in Table 1. In addition, the networks showing unit cell and intermolecular/intramolecular hydrogen bonds are given in Figure 3 and Figure 4. The intra-intermolecular interactions are listed in Table 2 [41]. Accordingly, there are intramolecular hydrogen bond interaction between O1-H1∙∙∙∙∙∙N3 atoms with the distance of 1.907 Å. The structure includes a thiosemicarbazone cation with one counter bromide ion. The structure of the thiosemicarbazone contain hydrogen-bromide bonding interactions between bromide ions and the H1A and H1C atoms of ammonium group of the cationic moiety with the bond distances 2.634 and 2.673 Å for N1-H1C∙∙∙∙∙Br1 and N1-H1A∙∙∙∙∙Br1, respectively.

### 3.4. Antioxidant properties analysis

**Figure 5:**
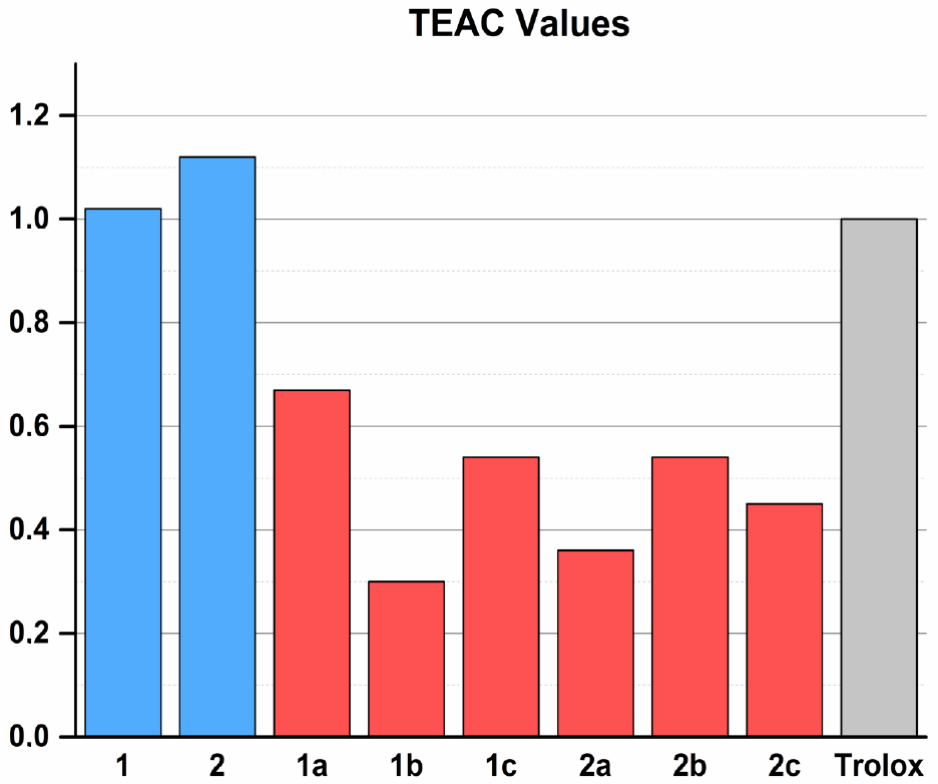
TEAC values of the compounds..

The CUPRAC method [26] was applied to N^1^-4-benzyloxysalicylidene-S-methyl/propyl-thiosemicarbazones and nickel(II) complexes in comparison with the trolox standard reference compound. The linear equations (A = mC + n), correlation coefficients (r), and linear concentration ranges of tested compounds summarized in Table 3. The TEAC values of the ligands and complexes can be seen in Figure 5. Their trolox equivalent antioxidant capacities (TEAC coefficients) were calculated from the ratio of the slope of the calibration curve of each compounds to that of trolox. 

**Table 3:** The TEAC (trolox equivalent antioxidant capacity) coefficients of the compounds with respect to the CUPRAC assay.

Sample	Working range	Calibration equation	Correlation coefficient (r)	TEAC
1	6.1 × 10–6 – 3.0 × 10–5	A = 17034 c + 0.130	0.9918	1.02 ± 0.05
2	6.1 × 10–6 – 3.0 × 10–5	A = 18644 c + 0.119	0.9894	1.12 ± 0.01
1a	1.1 × 10–5 – 5.5 × 10–5	A = 11242 c + 0.165	0.9946	0.67 ± 0.03
1b	1.2 × 10–5 – 6.1 × 10–5	A = 5098 c + 0.052	0.9956	0.30 ± 0.04
1c	1.1 × 10–5 – 5.5 × 10–5	A = 9166 c + 0.046	0.9933	0.54 ± 0.01
2a	1.1 × 10–5 – 5.5 × 10–5	A = 6008 c + 0.017	0.9880	0.36 ± 0.04
2b	1.1 × 10–5 – 5.5 × 10–5	A = 9067 c - 0.007	0.9976	0.54 ± 0.06
2c	6.1 × 10–6 – 3.0 × 10–5	A = 7604 c - 0.019	0.9923	0.45 ± 0.02

Trolox εN: 1.67 x 104 L mol–1 cm–1

The TEAC coefficients of the ligands were 1.02 and 1.12, as 1 and 2, respectively. The TEAC coefficients of the Ni(II) complexes with methyl derivatives were 0.67, 0.30 and 0.54, as 1a, 1b and 1c, respectively. The TEAC coefficients of the Ni(II) complexes with propyl derivatives were 0.36, 0.54 and 0.45, as 2a, 2b and 2c, respectively. When the synthesized compounds were compared with trolox as reference, the TEAC values of thiosemicarbazones (1 and 2) were higher than trolox (TEAC_trolox_=1.0). The nickel(II) complexes (1a-c and 2a-c) exhibited less antioxidant activity with lower TEAC coefficients. Also, when the TEAC values are examined, it is seen that the thiosemicarbazones are about twice as high as their antioxidant capacities compared to its nickel complexes. The position of the methoxy group in the benzene ring in nickel complexes affects the antioxidant capacity. When we examine the methyl derivatives of nickel(II) complexes, the high antioxidant capacity was obtained when the methoxy group was in 3th position, on the other hand, when we examined propyl derivatives, it was in the 4th position of the methoxy group.

Hydroxyl radicals were produced by reacting the iron-EDTA complex with H_2_O_2_. Hydroxyl radicals attack salicylate under acidic conditions, yielding a yellow product with Cu(II) -Nc. The added radical scavengers compete with salicylate for the produced hydroxyl radical and reduce chromophore formation from Cu(II)-Nc. HRS activity as inhibition ratio of the tested compounds was summarized in Table 4. When the HRS activities of the tested compounds were examined, it was found that there was no significant difference between the thiosemicarbazones and nickel complexes. On the other hand, the highest HRS activity belongs to 1 with 63.77% and the lowest belongs to 2b with 51.14%.

DPPH scavenging activities of the tested compounds also represent free radical scavenging activities. When Table 4 was examined, it can be seen that DPPH scavenging activities of propyl derivatives of Ni(II) complexes are higher than their methyl derivatives and free thiosemicarbazones. The highest DPPH scavenging activity belongs to 2c with 61.52%.

The ABTS radical scavenging activities of the tested compounds are summarized in Table 4 as percentage inhibition rate. ABTS radical scavenging activities of thiosemicarbazones were significantly higher compared to nickel(II) complexes. The results of ABTS radical scavenging activities of tested compounds were parallel with the antioxidant capacity results in view of ranking. Since ABTS radical scavenging activity also uses for total antioxidant capacity determination in the literature, it confirms the parallelism between CUPRAC results and ABTS radical scavenging activities obtained in the study. The highest ABTS scavenging activity belongs to 2 with 70.89% and the lowest belongs to 2a with 21.98%.

## 4. Conclusion 

Six new nickel(II) template complexes with N_2_O_2_ chelating thiosemicarbazones were synthesized with appreciable yield of between 51%–72%. The structural characterizations of all compounds were carried out by using analytical and spectroscopic methods. The structure of N^1^-4-benzyloxysalicylidene-S-methylthiosemicarbazone (2) was confirmed by X-ray diffraction method. The compounds were investigated for the effect of alkyl chain and the position of the methoxy group of the second aldehyde on antioxidant activity. The thiosemicarbazones exhibited more potent antioxidant capacity than nickel(II) complexes. This is probably due to the fact that thiosemicarbazones have non-coordinated -NH_2_ and -OH groups, which allow the coordination of Cu(II) ion in CUPRAC method. With the formation of the complex, the thiosemicarbazones have coordinated with the nickel(II) ion via these groups. Thus, the antioxidant activity of the nickel(II) complexes is lower as expected. In the S-methyl derivative complexes, the high antioxidant capacity was obtained in the 1a where the methoxy group is in the 3th position. However, when S-propyl derivative complexes were examined for radical scavenging activity, highest activity was obtained in the 2b where the methoxy group is in the 4th position. In addition, it was observed that the radical scavenging activities of thiosemicarbazones were mostly higher than Ni(II) complexes, except DPPH activities. As can be seen, the synthesis of thiosemicemicarbazone derivatives and metal complexes with different substitutions plays an important role in obtaining the desired structure.
